# Supplementary material for: Surface Microbiome Profiling of Dental Elevators Using Third‐Generation Sequencing: Implications for Infection Control in Dental Practice
Source: Microbiologyopen. 2025 Dec 8;14(6):e70178. doi: 10.1002/mbo3.70178 (PMC12685768; doi:10.1002/mbo3.70178)
Supplement: Supplementary file 1 — customer_backup. [file MBO3-14-e70178-s001.zip › customer_backup/customer_backup/taxa_summary/krona/samples/M08.Krona.html]

Javascript must be enabled to view this page.

members
magnitude
magnitudeUnassigned

M08.krona

48350

48330

3784

2713

2713

13

13

13

168

4

4

1

1

1

1

144

143

1

8

8

1

1

9

9

2532

178

146

32

2354

221

1939

188

6

7

2

2

2

2

2

1

1

1

1

1

1

3

3

3

3

136

135

2

2

2

133

133

133

1

1

1

1

926

97

97

1

1

96

5

2

10

5

5

38

31

1

1

1

1

153

2

1

1

1

1

151

70

70

63

63

6

6

12

12

9

8

8

8

1

1

1

7

7

7

4

2

1

551

551

459

459

78

11

22

14

28

1

2

14

14

108

1

1

1

29

25

25

3

3

1

1

1

1

1

76

45

45

3

3

3

3

1

1

24

24

1

1

1

2

2

2

2

2

1193

1193

1193

1193

1193

254

215

21

165

219

83

236

68

68

68

68

68

68

3805

3805

3805

3805

3805

180

3616

9

10106

10106

10106

9900

1

1

9899

8765

1134

206

206

26

28

40

10

14

21

18

49

1

1

1

1

1

1

4

4

4

1

1

1

1

1

1

1

1

1

1

1

1

57

16

16

16

12

12

4

4

1

1

1

1

1

40

40

40

40

40

3

1

1

1

1

1

2

2

2

2

2

645

6

6

6

6

6

639

639

400

54

54

33

33

258

258

55

55

239

239

239

1

1

1

1

1

1

43

38

38

38

38

38

5

5

5

5

5

424

424

424

424

379

140

174

65

45

45

62

62

62

62

62

62

2466

385

43

43

43

43

54

54

54

54

1

1

1

1

2

2

1

1

1

1

7

7

1

1

1

1

5

2

1

1

1

118

118

1

1

38

38

79

78

1

64

30

13

13

3

3

1

1

9

9

4

3

1

3

1

1

2

2

3

3

3

28

16

15

1

12

12

91

91

91

91

5

5

2

2

1

1

2

2

2081

1019

61

61

61

3

2

1

1

1

1

4

4

4

54

5

5

42

42

7

7

21

16

16

3

3

2

2

469

205

205

19

7

12

160

160

1

1

84

84

407

320

320

87

1

85

1

692

4

2

2

2

2

2

2

2

3

3

3

619

579

579

4

4

36

36

2

2

1

1

58

4

4

26

2

24

28

28

4

4

4

1

1

1

1

13

13

13

3

10

1

1

1

1

135

135

135

81

54

186

186

186

186

34

1

1

1

15

15

14

1

18

9

8

1

8

8

1

1

15697

8168

1

1

1

1

308

308

308

308

77

77

70

70

7

7

13

13

13

13

2517

2291

9

9

1

1

266

252

2

12

148

41

107

53

52

1

1

1

4

2

2

2

2

1

1

6

6

18

18

79

67

6

1

5

10

10

41

41

44

43

1

130

4

4

111

11

57

1

49

7

22

2

2

16

2

22

22

7

7

158

158

22

22

29

29

1

1

28

23

5

74

74

5

5

59

11

48

40

40

6

6

1

1

172

9

163

539

3

153

298

33

52

1

1

235

165

70

226

226

226

4339

867

75

75

98

9

89

334

293

41

261

261

77

71

6

22

22

2705

1

1

1473

1473

36

36

1173

1173

22

22

767

767

767

4

4

4

3

1

12

9

9

9

3

3

3

859

644

1

1

206

3

1

202

34

34

3

1

2

55

55

4

4

91

91

2

2

248

1

247

25

25

25

162

1

1

158

158

2

2

1

1

28

28

28

38

1

1

1

15

15

15

22

22

22

4785

226

94

91

91

2

1

1

1

1

132

132

2

2

1

17

7

2

101

1011

611

94

94

6

2

1

3

504

504

2

2

5

5

400

16

16

21

21

122

122

40

40

12

12

1

1

10

10

3

3

2

2

39

39

34

34

100

100

102

15

8

8

7

2

5

87

87

87

1

1

1

1

4

1

1

1

3

3

3

1

1

1

1

3168

6

6

5

1

10

7

7

3

3

2319

2201

66

6

1353

14

4

758

118

8

110

6

6

1

5

825

1

1

1

1

13

8

5

11

8

3

134

103

2

29

9

9

339

14

1

319

5

20

20

283

238

12

33

7

5

2

4

4

3

3

2

2

2

258

258

258

62

45

151

14

14

1

1

13

13

2744

2744

954

419

1

418

535

109

93

60

15

79

24

22

133

1790

59

52

7

704

704

988

4

340

552

92

39

35

4

4

4

4

4

4

4

11

11

11

11

11

11

9956

9956

1

1

1

1

669

664

664

2

50

555

57

4

4

4

1

1

1

9176

4

4

4

8

8

8

10

10

10

77

26

26

51

51

40

33

33

6

4

2

1

1

1078

1078

1078

72

72

72

1740

1740

573

1135

32

72

72

1

1

5

1

2

59

3

735

1

1

702

638

64

32

31

1

258

258

258

5082

526

41

23

1

461

2

2

1

1

2225

26

80

434

106

196

8

5

44

44

160

12

1092

13

5

2328

56

49

255

431

208

366

64

28

208

663

110

110

110

22

88

4

4

4

4

4

4

4

16

7

7

7

7

7

7

7

7

7

7

7

7

2

2

2

2

2

2
